# Synergistic effect of octenidine gel and hydrogel dressing in the treatment of venous leg ulcers

**DOI:** 10.3389/fphar.2026.1799111

**Published:** 2026-04-28

**Authors:** Ewa Rojczyk, Kinga Spyrka, Aleksander Sieroń, Dariusz Bazalińśki, Marek Kucharzewski

**Affiliations:** 1 Wladyslaw Bieganski Collegium Medicum, Jan Długosz University in Częstochowa, Częstochowa, Poland; 2 Faculty of Medicine, Academy of Silesia in Katowice, Katowice, Poland; 3 Institute of Nursing, Faculty of Health Sciences and Psychology, Collegium Medicum, University of Rzeszów, Rzeszów, Poland; 4 Laboratory for Innovative Research in Nursing, University Center for Research and Development in Health Sciences, Faculty of Health Sciences and Psychology, Collegium Medicum, University of Rzeszów, Rzeszów, Poland; 5 Department of General Surgery, Surgical Outpatient Clinic of Healthcare Centre of Jan Paweł II District Hospital in Włoszczowa, Włoszczowa, Poland

**Keywords:** dressing, healing, hydrogel, octenidine, venous leg ulcer, wound

## Abstract

**Introduction:**

Venous leg ulcers (VLUs) represent a major chronic wound condition associated with high recurrence rates, prolonged healing, and substantial socioeconomic burden. Hydrogels have recently gained attention as effective wound dressings due to their moisture-retentive and biocompatible properties. This study aimed to evaluate the clinical effectiveness of a combined therapy using octenidine gel and a hydrogel dressing compared to a silver-containing calcium alginate dressing in patients with VLUs.

**Methods:**

A randomized controlled trial was conducted, enrolling 60 patients with chronic VLUs. Participants were randomly assigned to either an experimental group (octenidine gel + hydrogel dressing) or a control group (silver-containing calcium alginate dressing). Wound healing progress was assessed using the RESVECH 2.0 Scoring Scale, which evaluates six wound parameters of effective wound healing. Measurements were taken at baseline and after 4 weeks of treatment.

**Results:**

After 4 weeks, the experimental group showed statistically significant improvements in wound size, wound edges, tissue in the wound bed, exudate, infection/inflammation signs, and overall RESVECH 2.0 total score. No significant changes were observed in the control group. Between-group comparisons confirmed superior healing outcomes in the experimental group.

**Discussion:**

The combination of octenidine gel and a hydrogel dressing significantly enhanced healing outcomes in patients with VLUs compared to conventional silver-containing dressings, suggesting that hydrogel-based antiseptic therapy is an effective alternative for VLU management. Given the chronic nature and high recurrence rates of VLUs, incorporating hydrogel-based antiseptic dressings into standard treatment protocols may improve patient outcomes and reduce healing time.

## 1 Introduction

Venous leg ulcers (VLUs) pose a significant public health problem worldwide. Data on their global prevalence and incidence remain limited, and published findings often vary due to differences in study design and measurement methods (Probst et al., 2023).

These ulcers develop on the lower limb and account for approximately 60%–80% of all leg ulcers (Körber et al., 2011; 2017). Their healing rate at 3 months is estimated at around 40% (Rajhathy et al., 2020), yet up to 80% of patients experience a recurrence within 3 months of healing (Abbade and Lastória, 2005). Reported prevalence rates are about 1.08% (Guest et al., 2020), while incidence can reach 1.33% (Margolis, 2020).

VLUs are a matter of concern both internationally and locally. Even with appropriate treatment, as many as 20% fail to heal within 2 years (Callam et al., 1987; Raju and Fredericks, 1990; Chaby et al., 2013). They place a substantial social and economic strain, with estimated annual costs of £102 million in the UK (Urwin et al., 2022), $32 billion in the United States (Nussbaum et al., 2018), and AUD $3 billion in Australia (Weller and Evans, 2012).

Risk factors for venous leg ulcers (VLUs) include older age, a history of deep vein thrombosis, being female, phlebitis, and excess body weight (Harris et al., 2017). With rising obesity rates and an aging population, the incidence of chronic wounds, including VLUs, is expected to reach epidemic levels (Apovian, 2016; Cereceres et al., 2019; Chooi et al., 2019). This condition significantly reduces patients’ quality of life. Many suffer from persistent pain, limited mobility, and difficulties with daily tasks such as dressing or bathing. Social interactions and mental health are also affected, with approximately 30% of patients experiencing anxiety or depression (Neumann et al., 2016; Harris et al., 2017).

The standard approach to VLU management involves local wound care combined with compression therapy (Aziz and Cullum, 2015; Sawad and Turkistani, 2020). Surgical and endovascular procedures designed to correct abnormal venous flow are also among the primary treatment options (Aziz and Cullum, 2015; Cuomo et al., 2017). Patient education plays a crucial role in successful therapy, focusing on self-care practices such as proper wound management, exercise, and diet.

Dressings are used in wound treatment to promote healing and reduce the risk of infection. They facilitate fluid absorption, support re-epithelialization, maintain an optimal level of moisture, and protect the wound from external factors. Available options include moist occlusive dressings, hydrogels, and semi-permeable films (Coalson et al., 2019; Sawad and Turkistani, 2020).

Hydrogels have gained attention as modern wound dressings for the management of both acute and chronic injuries, including burns, diabetic foot ulcers, venous ulcers, and pressure sores. These polymeric structures maintain a moist healing environment, support cell migration, and exhibit antimicrobial activity, making them more effective than traditional dressings (Alberts et al., 2025). Moreover, hydrogel dressings promote autolytic debridement and angiogenesis, while reducing pain during dressing changes (Zhang et al., 2019; Sopata et al., 2021).

Given the complexity of chronic wounds, hydrogels offer a promising therapeutic approach thanks to their compatibility with biological tissues, capacity to retain moisture, and structural adaptability. Nevertheless, future research should consider the multifaceted nature of wound sites, where factors such as pH imbalance, elevated reactive oxygen species (ROS), and unique enzyme activity play critical roles (Jia et al., 2023).

An advanced direction in hydrogel design involves multifunctional materials capable of accelerating healing, preventing infection, and enabling personalized therapy tailored to specific wound types. Recent investigations highlight this trend, with innovative hydrogel formulations incorporating antimicrobial components (such as silver nanoparticles, honey, and zinc-modified ceria), bioactive agents, and anti-inflammatory substances (Zhao et al., 2024; Bîrcă et al., 2025).

Hydrogel dressings, identified in the Nurse Prescribers’ Formulary as advanced wound dressings, are developed to create an optimal healing environment. They do this by donating moisture to dry, sloughy wounds and by encouraging autolytic debridement of necrotic tissue. Depending on the wound’s moisture balance, some hydrogels can also absorb small amounts of exudate or help rehydrate the wound (BMJ Group, n.d.). Certain types are combined with alginates (e.g., Nu-Gel, Purilon Gel) (BMJ Group, n.d.), which offer greater absorbency and support chemical debridement (Mandelbaum et al., 2003).

A key benefit of hydrogel dressings is their versatility, as they can be applied during different stages of wound healing. They also tend to provide comfort and may help ease pain in painful wounds, though they require a secondary dressing for secure application (Bradbury et al., 2008).

By adding moisture, hydrogels can rehydrate, soften, and break down non-viable tissue, thereby aiding in desloughing and potentially influencing ulcer healing. However, current evidence does not provide clear guidance on their effectiveness in treating VLUs or on whether they should be recommended for clinical or policy use (Palfreyman et al., 2007; Norman et al., 2018). Thus, despite the growing body of literature on the management of VLUs, more research is needed to compare the outcomes of hydrogel dressings with those of alternative dressing types for venous ulcers. Existing studies often differ in design, follow-up duration, and outcome measures, which limits the ability to draw clear clinical conclusions. Our study addresses these limitations by employing an uncommon comparative framework that evaluates two distinct and increasingly utilized treatment modalities.

Interestingly, the 2024 guideline by Valesky et al. (2024) underscores that, despite advances, VLU management still lacks high-quality evidence for many modern dressings and antiseptic strategies. Thus, by delivering robust randomized controlled trial data, our study helps to address this knowledge gap, potentially informing future guideline revisions.

The primary aim of this study was to compare the clinical effectiveness of two different dressings for VLUs: an octenidine-based gel combined with a hydrogel dressing versus a silver-containing calcium alginate dressing. Specifically, the study sought to evaluate and compare the wound healing progress achieved with each therapy over a 4 week treatment period using the RESVECH 2.0 scale. The secondary objectives were to assess changes in key wound parameters—including wound size, tissue type, exudate level, and signs of inflammation—and to determine whether the use of octenidine-based therapy could offer a clinically significant advantage in promoting healing and improving overall wound condition compared to the standard silver-based approach.

## 2 Materials and methods

### 2.1 Research group

The randomized controlled trial enrolled 60 patients diagnosed with venous leg ulcers, who were randomly allocated to either the experimental or control group. In the experimental group, treatment consisted of octenidine gel in combination with a hydrogel dressing, whereas patients in the control group received a silver-containing calcium alginate dressing. No formal *a priori* sample size calculation was performed. The sample size (n = 60) was determined pragmatically based on feasibility considerations and the number of eligible patients available during the study period.

Inclusion Criteria:

- A wound treated unsuccessfully for more than 3 months
- A wound area ranging from 20 to 60 cm^2^
- Patient’s signed informed consent to participate in the study

Exclusion Criteria:

- Allergy to silver or octenidine
- No informed consent provided

**FIGURE 1:**
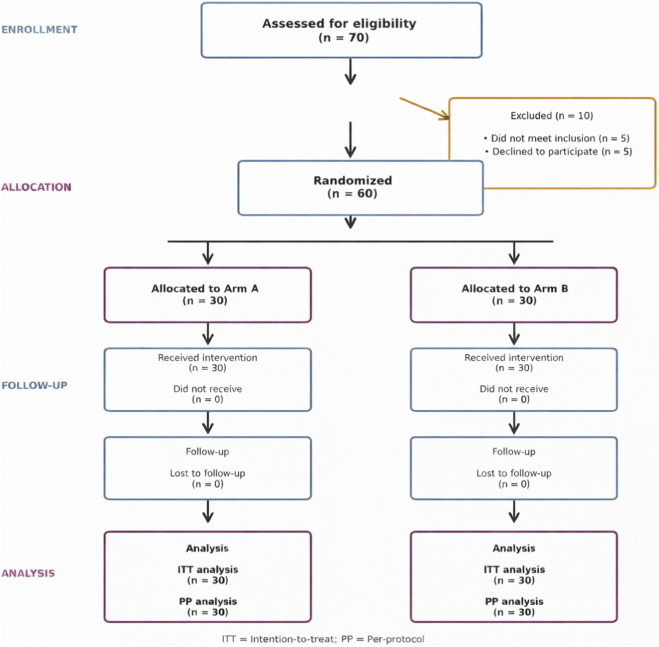
CONSORT flow diagram illustrating the process of participant screening, randomization, follow-up, and analysis. A total of 70 patients were evaluated for eligibility, of whom 10 were excluded (five did not meet the inclusion criteria and five declined to participate). Sixty participants were then randomly allocated in a 1:1 ratio to two groups (Arm A and Arm B; n = 30 per group). All enrolled participants received the allocated intervention and completed the follow-up period. No participants were lost to follow-up, and all were included in both the intention-to-treat (ITT) and per-protocol (PP) analyses.

A total of 70 individuals were assessed for eligibility based on the predefined inclusion and exclusion criteria. Of these, 10 were excluded—five did not meet the inclusion criteria and five declined to participate. The remaining 60 participants were randomly assigned in a 1:1 ratio to two study arms: Arm A (n = 30) and Arm B (n = 30). All randomized participants received the assigned intervention and completed the full follow-up period. No losses to follow-up were recorded, and all 60 participants were included in both the intention-to-treat and per-protocol analyses. A detailed overview of participant flow is presented in Figure 1.

Allocation was performed using a sealed-envelope randomization method. Each participant drew an opaque, sealed envelope indicating assignment to either the experimental or control group, thereby maintaining blinding with respect to group allocation. Due to the clearly distinguishable physical characteristics of the dressings, blinding of participants and treating clinicians was not feasible.

The study was conducted in Healthcare Centre of the Jan Paweł II District Hospital in Włoszczowa, and in the Wound Treatment Clinic of the Specialist Hospital of Priest B. Markiewicz, Subcarpathian Oncology Centre in Brzozowo.

Data collection was carried out between January and December 2025. All patients agreed to participate in the study by signing informed consent. The study was approved by the Ethics Committee of Jan Długosz University in Częstochowa (protocol code KB-U/1/2025) and was conducted in accordance with the Declaration of Helsinki.

### 2.2 Research tool

The effectiveness of the treatment was evaluated using the RESVECH 2.0 Scoring Scale (Table 1). This instrument assesses six key domains, yielding a total score ranging from 35 (indicating the most severe wound condition) to 0 (representing complete healing). Lower scores correspond to better wound status. The evaluated parameters include wound size, depth and tissue involvement, wound edges, tissue type in the wound bed, exudate, and signs of inflammation.

**TABLE 1:** RESVECH 2.0 scale–detailed breakdown of the scoring criteria for each feature.

Parameter	Score	Criteria
1. Wound size (area)	0	Healed (no wound)
	1	<4 cm^2^
	2	4–16 cm^2^
	3	16–36 cm^2^
	4	36–64 cm^2^
	5	≥64 cm^2^
2. Depth/Tissues involved	0	Skin intact
	1	Epidermis and dermis involved
	2	Subcutaneous tissue involved
	3	Muscle, tendon, or bone exposed
3. Wound edges	0	Well-defined, attached edges
	1	Diffuse or indistinct edges
	2	Detached or undermined edges
	3	Thickened, rolled, or hyperkeratotic edges
4. Tissue in the wound bed	0	100% epithelial tissue
	1	≥75% granulation tissue
	2	25%–74% granulation tissue
	3	<25% granulation tissue
	4	Slough or necrotic tissue present
5. Exudate	0	None
	1	Scant
	2	Moderate
	3	Heavy
	4	Copious or leaking
6. Infection/Inflammation signs	0–14	Each of the following signs scores 1 point: Increasing pain, perilesional erythema, perilesional edema, increased local temperature, increased exudate, purulent exudate, friable tissue, stagnant wound, tissue compatible with biofilm, odor, hypergranulation, increase in wound size, satellite lesions, pale tissue

The cumulative score across all six dimensions provides a comprehensive assessment of the wound’s condition, facilitating clinical monitoring of the healing process and enabling individualized adjustment of therapeutic interventions. The RESVECH 2.0 scale was administered twice—at baseline (prior to treatment initiation) and after a 4 week treatment period.

### 2.3 Research procedures

All patients underwent Doppler ultrasonography to evaluate lower-limb blood flow and to exclude ulcers of non-venous origin. Each patient was also thoroughly assessed with respect to the study’s inclusion and exclusion criteria. Prior to inclusion, ankle–brachial index (ABI) was measured in all patients to exclude significant arterial insufficiency. Only patients with ABI ≥ 0.8 were eligible for full compression therapy.

During dressing changes, patients were positioned on an examination couch in a semi-upright or semi-seated posture, in a room maintained at 23 °C and 40% relative humidity. In both study groups, wound cleansing was performed using an antiseptic solution containing 0.1% polyhexanide and poloxamer 188, followed by gentle mechanical debridement with a sterile Volkmann spoon.

Subsequently, the appropriate dressing was applied according to the group allocation. In the experimental group, a small amount of octenidine gel (Octenilin® Wound Gel, Schülke & Mayr GmbH, Norderstedt, Germany) was applied to the hydrogel dressing (Aqua-Gel®, Kikgel, Ujazd, Poland) and then it was placed directly on the wound. The control group received a calcium alginate dressing containing silver (Suprasorb® A + Ag, Lohmann and Rausche, Pabianice, Poland) which was also placed directly on the wound.

Then, the dressings in both groups were covered with sterile secondary dressings (gauze pads) and secured with a short-stretch, non-elastic compression bandage. For the proper measurement of compression pressure, the Kikuhime device pressure sensor was used and a pressure of 25–35 mm Hg was applied (corresponding to Class II compression).

Thus, all patients included in the study received standardized compression therapy, in accordance with current evidence-based recommendations for VLUs management. Compression was applied by trained medical personnel using a standardized technique and was maintained throughout the study period, with regular adjustments during follow-up visits. Patient adherence and tolerance were monitored at each visit. Importantly, the compression regimen was identical in both groups, ensuring that any observed differences in healing outcomes were attributable exclusively to the type of local wound therapy (octenidin vs. calcium alginate).

In both groups, dressings were replaced every 3 days under the same outpatient clinical conditions. The wounds were monitored over a period of 35 days.

### 2.4 Statistical analysis

Statistical analysis of the collected data was conducted using StatSoft Statistica 13.3. Both parametric and nonparametric tests were applied, with the selection of nonparametric methods based on the failure to meet the fundamental assumptions of parametric testing—specifically, the requirement of normal distribution of the examined variables, which was assessed using the Shapiro–Wilk test. Descriptive statistics, including mean and standard deviation, were calculated for all variables.

To evaluate differences in mean values between two independent groups, the Student’s t-test for independent samples or, when necessary, the nonparametric Mann–Whitney U test was used. Intragroup changes over time were assessed with the Wilcoxon signed-rank test. Qualitative variables were analyzed using Pearson’s chi-square test. A significance level of p < 0.05 was adopted for all statistical procedures. An approximate effect size (Hedges’ g) was estimated using the pooled standard deviation from pre- and post-treatment values.

## 3 Results

### 3.1 Anthropometric data

The study enrolled 60 patients, including 29 women (48.3%) and 31 men (51.7%). Participants were between 50 and 78 years of age, with a mean age of 61.88 ± 6.96 years. Wound areas ranged from 20.5 to 59.1 cm^2^, and wound duration varied from 3 to 12 months. The Body Mass Index (BMI) of participants ranged from 22 to 31 kg/m^2^, while Ankle–Brachial Index (ABI) values were between 0.91 and 1.1. No statistically significant differences were observed between the study groups with respect to these parameters (p > 0.05) (Table 2).

**TABLE 2:** Basic anthropometric and clinical features of patients (n = 30 per group).

Parameter	Experimental group	Control group	Significance
Gender			
Females	14 (46.7%)	15 (50.0%)	p = 0.796
Males	16 (53.3%)	15 (50.0%)	
Age [years]	62.27 ± 7.89	61.50 ± 5.99	t = 0.42
	(50–78)	(51–78)	p = 0.673
Wound area [cm^2^]	38.12 ± 12.42	35.94 ± 10.44	p = 0.52
	(20.5–59.1)	(20.5–57.4)	
Wounding time [months]	6.43 ± 1.99	6.07 ± 1.98	t = 0.71
	(3–12)	(3–10)	p = 0.478
Body Mass index (BMI) [kg/m^2^]	26.00 ± 2.32	26.13 ± 2.15	t = 0.23
	(22–31)	(23–31)	p = 0.818
Ankle brachial index (ABI)	0.95 ± 0.05	0.96 ± 0.04	p = 0.225
	(0.91–1.10)	(0.91–1.10)	

t – Student’s t-test value; p - test probability index (p-value).

Parameters are presented as number of patients (percentage) or mean value ±SD (range).

### 3.2 Clinical outcomes and representative case of wound healing with octenidin gel

**FIGURE 2:**
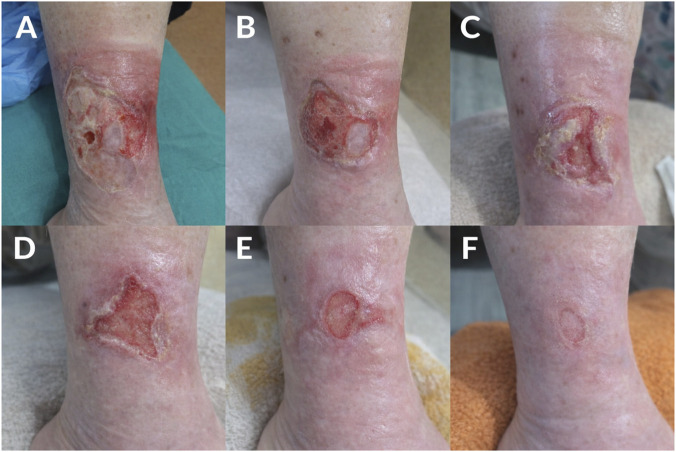
Clinical progression of wound healing in a 68-year-old female patient (undergoing treatment with octenidin gel) suffering from chronic venous leg ulcer (VLU) persisting for 2 years. The images **(A–F)** document the wound healing process at 7-day intervals. **(A)** Baseline presentation: extensive ulceration with fibrinous slough, moderate exudate, and surrounding erythema. **(B)** Day 7: partial reduction of fibrin and increased granulation tissue formation. **(C)** Day 14: further debridement effect with visible granulation and reduced inflammatory signs. **(D)** Day 21: progressive wound contraction and predominance of healthy granulation tissue. **(E)** Day 28: marked epithelialization with minimal residual defect. **(F)** Day 35: near-complete healing with restoration of skin integrity.

Figure 2 illustrates representative outcome of treatment in the experimental group at six different time points. The clinical progression of wound healing is demonstrated in a 68-year-old female patient with a chronic VLU persisting for 2 years, treated with octenidin gel. At baseline, the ulcer presented as an extensive lesion covered with fibrinous slough, accompanied by moderate exudate and surrounding erythema. After 7 days of treatment, a partial reduction in fibrin deposition was observed, along with the initiation of granulation tissue formation. By day 14, further debridement effects were evident, with more pronounced granulation tissue and a noticeable reduction in inflammatory signs. At day 21, continued wound contraction was observed, with predominance of healthy granulation tissue within the wound bed. By day 28, marked epithelialization was noted, leaving only a minimal residual defect. At the final assessment on day 35, near-complete healing was achieved, characterized by restoration of skin integrity.

### 3.3 Absolute scores on the REVESTECH 2.0 scale

The effectiveness of the treatment was determined based on the RESVECH 2.0 scores obtained by the subjects within 4 weeks of treatment initiation.

Significant differences between the initial and final measurements were demonstrated in the experimental group for the following parameters: Wound Size (Area) (p = 0.017), Wound Edges, Tissue in the Wound Bed, Exudate, Infection/Inflammation Signs and the Total Score (p ≤ 0.001). In each of the above-mentioned categories, the scores in RESVECH 2.0 scale after 4 weeks of treatment were lower compared to the initial measurement, i.e., wound condition improvement was observed. Only in case of Depth/Tissues Involved category, there were no differences between absolute values of points in two timepoints studied. In the experimental group, the reduction in the total RESVECH 2.0 score was associated with a very large effect size (Hedges’ g = 3.26). Very large effects were also observed for Infection/Inflammation Signs (g = 3.57) and Exudate (g = 2.47), whereas the effect for Wound Size was small to moderate (g = 0.36) (Table 3).

**TABLE 3:** RESVECH 2.0 parameters (points in RESVECH 2.0 scale) before treatment and after 4 weeks of treatment (n = 30 per group) in experimental and control group.

Parameter	Group	Before (mean ± SD)	After 4 weeks (mean ± SD)	% Change over 4 weeks>	p-value	Hedge’s g-value
Wound size (area)	Experimental	3.50 ± 0.51	3.27 ± 0.74	−6.6%	0.017	0.36
	Control	3.47 ± 0.51	3.47 ± 0.51	0.0%	-	≈0
Depth/Tissues involved	Experimental	2.00 ± 0.00	2.00 ± 0.00	0.0%	-	≈0
	Control	2.00 ± 0.00	2.00 ± 0.00	0.0%	-	≈0
Wound edges	Experimental	1.00 ± 0.00	0.53 ± 0.51	−47.0%	<0.001	1.29
	Control	1.00 ± 0.00	1.00 ± 0.00	0.0%	-	≈0
Tissue in the wound bed	Experimental	2.47 ± 0.51	2.03 ± 0.18	−17.8%	0.001	1.14
	Control	2.60 ± 0.50	2.60 ± 0.51	0.0%	-	≈0
Exudate	Experimental	2.73 ± 0.64	1.27 ± 0.52	−53.4%	<0.001	2.47
	Control	2.60 ± 0.62	2.60 ± 0.72	0.0%	1.000	≈0
Infection/Inflammation signs	Experimental	6.13 ± 0.97	2.73 ± 0.91	−55.5%	<0.001	3.57
	Control	6.40 ± 1.25	6.17 ± 1.34	−3.6%	0.061	0.18
Total score	Experimental	17.83 ± 1.98	11.83 ± 1.64	−33.6%	<0.001	3.26
	Control	18.07 ± 2.45	17.83 ± 2.59	−1.3%	0.142	0.09

SD, standard deviation; p - test probability index (p-value); p < 0.05 is considered statistically significany; Hedge’s g - approximate effect size estimated using the pooled standard deviation from pre- and post-treatment values; % change refers to the change in the mean values of points on the RESVECH 2.0 scale over 4 weeks of treatment.

In contrast, in the control group, there were no statistically significant differences in the RESVECH 2.0 scale scores obtained before treatment and after 4 weeks of treatment. For anatomical features like Wound Size, Depth/Tissue Involved and Wound Edges as well as for Tissue in the Wound Bed and Exudate categories, there were even no differences between absolute values of points in these two timepoints. Interestingly, Infection/Inflammation Signs parameter and Total Score on the RESVECH 2.0 scale improved, which can be concluded from the lower scores after 4 weeks of therapy. Hovewer, these changes were small (−3.6% and −1.3%, respectively) and statistically insignificant. Effect sizes in the control group were also negligible for most outcomes, including the total RESVECH 2.0 score (g = 0.09) (Table 3).

### 3.4 Changes of scores on the REVESTECH 2.0 scale after 4 weeks long treatment

**FIGURE 3:**
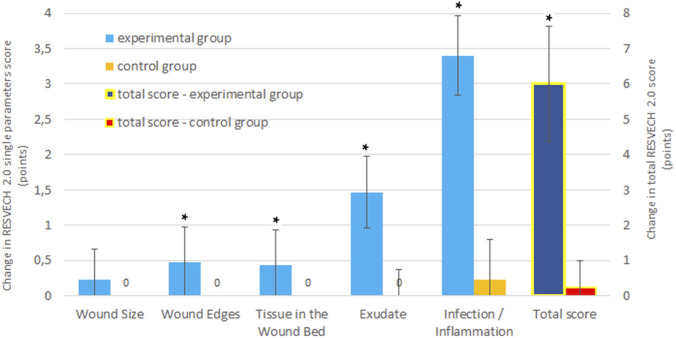
Changes in individual RESVECH 2.0 parameters and total score after 4 weeks of treatment in the experimental and control groups. Bars represent mean change (in points) for each parameter. Component scores are presented on the primary y-axis, while the total RESVECH 2.0 score is displayed on the secondary y-axis due to its different value range. Positive values indicate improvement in wound healing. Statistical significance is indicated where applicable. * - p < 0.01 vs. control group.

The effects obtained in the experimental and control groups over the 4-week treatment period were compared. The treatment effect was significantly greater in the experimental group (vs. control group) for most of the parameters studied: Wound Edges, Tissue in the Wound Bed, Exudate and Infection/Inflammation Signs. Bars for control group are mostly not visible, as the value was different than 0 only for one parameter–Infection/Inflammation Signs (0.23 ± 0.77). Data for Depth/Tissues Involved category is not shown, because all values are 0 which indicates that there were no differences in RESVECH 2.0 scores after treatment in either the experimental or control group (Figure 3).

The total score (sum of points on the REVESTECH 2,0 scale) remained unchanged in the control group, whereas in the experimental group it was significantly reduced 4 weeks after treatment compared with baseline (Table 3). This translates into a statistically significant change in the Total Score on RESVECH 2.0 scale in the experimental group compared to the control group (Figure 3).

## 4 Discussion

Recent years have seen a growing body of evidence evaluating advanced wound dressings in the management of venous leg ulcers (VLUs). A comprehensive meta-analysis published in 2024, synthesizing data from 18 randomized trials, demonstrated that although silver-containing dressings significantly improved healing rates in diabetic foot ulcers, their effectiveness in VLUs did not reach statistical significance, suggesting limited and inconsistent benefits in this patient population (Yi et al., 2024). In contrast, the most recent reviews from 2023 to 2024 highlight the rapid development of hydrogel-based wound therapies, particularly formulations enriched with antimicrobial or anti-inflammatory agents, which offer enhanced moisture balance, support autolytic debridement, and modulate local inflammation (Liang et al., 2024).

Additional experimental studies published in 2024 report that hydrogels incorporating antibiofilm and antioxidative functions promote faster healing and greater reduction of bacterial burden in chronic wounds than conventional dressings (Pranantyo et al., 2024). Clinical reports from recent years further emphasize that octenidine remains a safe and effective antiseptic option for chronic wounds, with demonstrated antibiofilm activity and no detrimental effects on tissue regeneration (Nair et al., 2023).

These observations are consistent with the results of our study, in which the combination of octenidine gel and a hydrogel dressing provides a significantly greater improvement in the healing of VLUs compared to the use of a silver-containing calcium alginate dressing. After 4 weeks, statistically significant improvements were observed in all six domains of the RESVECH 2.0 scale, indicating comprehensive enhancement of wound repair.

The use of the RESVECH 2.0 scale provided a multidimensional assessment of healing progress. Its validity and reliability have been confirmed in multiple cultural adaptations and wound types (Rodrigues et al., 2022; Menegon et al., 2023). The consistent improvement across all RESVECH 2.0 parameters in our experimental group underscores that octenidine-hydrogel therapy benefits not only wound contraction but also tissue regeneration, exudate management, and infection control.

Our results are consistent with prior studies showing the clinical benefits of octenidine formulations in chronic wound management. Hämmerle and Strohal reported superior wound-size reduction and cost-effectiveness of octenidine wound gel compared to phase-adapted standard dressings in VLUs over 42 days (Hämmerle and Strohal, 2014). Similarly, Braun et al. demonstrated that Octenilin® (an octenidine-containing product) improved wound bed condition, stimulated granulation tissue, and reduced treatment costs in chronic wounds (Braun and Price, 2014).

In a multicentre, open-label, post-marketing randomized follow-up study, Stryja et al. (2023) evaluated the clinical efficacy and safety of a dressing combining octenidine and hyaluronan compared to a standard silver-containing dressing in “hard-to-heal” wounds. Observed faster reduction of wound area and better skin/tissue condition in octenidine-treated group confirm our findings in a VLU-specific population, strengthening external validity of our results across different chronic wound types. Moreover, in case series from 2023, Nair et al. (2023) reported on four patients with chronic, hard-to-heal wounds treated with octenidine-based products. Among them, two had VLUs and two had diabetic foot ulcers (DFUs). This case series provides real-world, clinical-practice confirmation of tolerability and potential efficacy of octenidine-based therapy in VLUs.

Collectively, above-mentioned findings align with the current study, suggesting that octenidine’s broad-spectrum antimicrobial activity and low cytotoxicity support effective wound decontamination while preserving viable tissue.

In contrast, silver-containing dressings, although widely used for chronic wound management, have demonstrated variable clinical efficacy. Cochrane reviews by O’Meara et al. (2015) and Norman et al. (2018) indicated limited and inconsistent evidence regarding the superiority of silver-based or alginate dressings for VLU healing compared with non-antimicrobial alternatives. Similarly, more recent randomized trials, such as Beraldo et al. (2025) have shown that silver dressings may not significantly accelerate healing in “hard-to-heal” venous ulcers, especially when bacterial load is moderate. These findings parallel the current study’s observation that the silver-alginate control group exhibited no significant improvement in total RESVECH 2.0 scores after 4 weeks.

The improved healing in the experimental group likely reflects the synergistic effects of octenidine’s antimicrobial action and the hydrogel’s moisture-retentive environment. Ousey et al. (2016) emphasized the critical role of hydration in wound repair, demonstrating that balanced moisture supports keratinocyte proliferation and extracellular matrix remodeling. Similarly, Zhang et al. (2019) and Hosseini et al. (2025) confirmed in systematic reviews, that hydrogel dressings significantly improve healing rates in chronic wounds by maintaining physiological moisture and minimizing pain during dressing changes. Furthermore, *in vitro* studies show that hydrogel biomaterials may modulate autophagy and extracellular matrix remodeling (Li et al., 2021), mechanisms that could explain the improved tissue quality and wound-bed parameters observed in the present study.

While the short-term outcomes of this study are promising, several contextual factors merit consideration. As highlighted in systematic reviews (Norman et al., 2018; Shaydakov et al., 2022), chronic venous ulcers are multifactorial conditions influenced by venous insufficiency, infection, comorbidities, and adherence to compression therapy. The synergistic benefits observed with octenidine + hydrogel dressing likely complement, but do not replace the essential role of compression in achieving sustained healing. Fortunately, although our follow-up period was limited to 4 weeks, evidence from long-term studies (Hämmerle and Strohal, 2014) suggests that octenidine-based therapies can maintain healing benefits and reduce recurrence risk over extended periods.

From a mechanistic standpoint, the enhanced healing observed with octenidine + hydrogel therapy can be attributed to several factors. Octenidine exhibits rapid bactericidal activity against Gram-positive and Gram-negative organisms, including biofilm-forming species commonly present in VLUs. Kramer et al. (2018) emphasized octenidine’s favorable safety profile and low propensity to induce resistance, making it suitable for repeated application on chronic wounds. When integrated into a hydrogel, octenidine can be gradually released, maintaining prolonged antimicrobial activity at the wound surface while avoiding cytotoxic damage seen with other antiseptics.

Consequently, from the clinical point of view, the use of hydrogel dressings containing octenidine can be considered a promising adjunct to standard compression therapy in the management of VLUs. Their application may accelerate healing, reduce infection risk, and decrease the frequency of dressing changes, thereby improving patient comfort and adherence to therapy. Incorporating such advanced materials into evidence-based wound care protocols may contribute to optimizing resource use and achieving better clinical outcomes in chronic wound management. In practical terms, the integration of octenidine-hydrogel dressings into standard VLU care protocols could improve healing trajectories while reducing treatment costs and dressing-change frequency. The favorable patient tolerance and reduced pain reported in prior studies (Braun and Price, 2014; Sopata et al., 2021) further support the clinical feasibility of this approach.

### 4.1 Limitations of the study and future perspectives

However, despite promising results, the study’s limitations should be acknowledged.

First, the relatively small sample size (n = 60) and single-center design may limit the generalizability of the results to broader populations with VLUs of varying etiologies, comorbidities, and clinical settings. The absence of a formal *a priori* power calculation limits the ability to determine whether the study was adequately powered to detect clinically meaningful differences between groups.

Secondly, the follow-up period of 4 weeks, although sufficient to observe early wound healing dynamics, was insufficient to assess long-term outcomes such as complete ulcer closure, recurrence rates, or sustained improvements in skin integrity. This relatively short observation window limits the interpretation of the durability and clinical significance of the intervention, particularly in the context of VLUs, which are characterized by chronicity and typically require extended healing periods. Consequently, the findings should be interpreted as reflecting early treatment responses rather than definitive evidence of long-term effectiveness. However, although the follow-up period was limited to 4 weeks, this time frame is clinically relevant for the early assessment of local wound therapy, as lack of improvement within 2–4 weeks generally indicates the need for reassessment and possible modification of the treatment strategy.

Moreover, although randomization minimized selection bias, blinding of patients and clinical evaluators was not feasible due to the distinct physical properties of the dressings, which made group allocation readily identifiable during treatment and assessment. As a result, outcome evaluation using the RESVECH 2.0 scale was not performed in a blinded manner. Given that this tool relies on clinical judgment and visual inspection, the lack of assessor blinding may have introduced observer bias and potentially influenced the scoring of wound healing parameters.

Finally, confounding factors such as differences in patient adherence to compression therapy, nutritional status, and systemic health conditions were not exhaustively controlled for and could have influenced the healing process.

Future research should aim to validate these findings in larger, multicenter trials with extended follow-up periods (e.g., 12–24 weeks) and appropriate power calculations to determine the long-term efficacy and recurrence prevention potential of octenidine–hydrogel therapy as well as to provide a more comprehensive evaluation of clinical outcomes. Comparative studies evaluating cost-effectiveness, patient-reported outcomes, and quality of life measures are warranted to better establish the clinical and economic advantages of this approach.

Future studies should also consider incorporating blinded outcome assessment, for example, through independent evaluators or standardized photographic documentation, to reduce the risk of bias. They should also incorporate hard clinical endpoints such as complete wound closure rates, time to healing, and recurrence, in accordance with international wound care recommendations. Importantly, mechanistic investigations into the molecular and cellular effects of octenidine within hydrogel matrices could provide valuable insights into its antimicrobial and tissue-regenerative properties.

Exploring combination protocols with compression therapy optimization or adjunctive modalities such as negative pressure wound therapy may further enhance healing outcomes. Finally, standardized wound assessment tools and digital monitoring technologies could improve the objectivity and reproducibility of future evaluations.

### 4.2 Conclusions

To sum up, our findings support the growing evidence that hydrogel-based antiseptic therapies represent a valuable alternative to conventional silver-based dressings for chronic venous ulcers, which are difficult to heal and highly recurrent wounds. We provide important evidence highlighting the relative clinical utility of these therapies, which contributes to a more precise understanding of their effectiveness in the management of VLUs.

The results of this randomized controlled trial suggest that the combination of octenidine gel and a hydrogel dressing may improve the healing of VLUs compared with a silver-containing calcium alginate dressing. After 4 weeks of therapy, patients treated with the octenidine–hydrogel combination demonstrated greater reductions of RESVECH 2.0 scale scores, indicating more favorable early changes in wound healing dynamics and tissue conditon. These findings may indicate that hydrogels (when employed as carriers for antiseptic agents such as octenidine) can provide a dual therapeutic effect by offering antimicrobial protection while supporting a moist wound environment conducive to epithelialization and debridement. However, these observations relate to short-term outcomes and should not be interpreted as evidence of long-term efficacy.

Nevertheless, given the chronic and recurrent nature of VLUs, the integration of hydrogel-based antiseptic dressings into routine clinical practice may represent an effective and well-tolerated strategy for improving treatment outcomes and patient quality of life. Importantly, further, large-scale, multicenter studies are recommended to confirm these results and to evaluate their long-term efficacy, cost-effectiveness and recurrence prevention potential.

## Data Availability

The raw data supporting the conclusions of this article will be made available by the authors, without undue reservation.
